# Application of a downward tract adherence method in the manual bedside placement of post-pyloric tubes in the intensive care unit

**DOI:** 10.3233/THC-230824

**Published:** 2024-07-12

**Authors:** Zanhua Zhang, Fang He, Zhebing Lin, Zhisu Li, Fei Xiang, Weiwei Cai

**Affiliations:** Intensive Care Unit, The Second Affiliated Hospital of Wenzhou Medical University, Wenzhou, Zhejiang, China

**Keywords:** Blind bedside, critical care patients, enteral nutrition, insertion skill, nasojejunal tube

## Abstract

**BACKGROUND:**

At present, there are few studies on the technical requirements of manual bedside placement of post-pyloric tube in Intensive Care Unit patients.

**OBJECTIVE:**

To investigate the application value of downward tract adherence method in the manual bedside placement of jejunal tubes.

**METHODS:**

In the downward group, 160 patients underwent manual bedside placement of jejunal tubes by a downward tract adherence method. In the conventional group, 144 patients were treated with conventional gas injection during the placement. The success rate, average time, and adverse reactions of the placement in the two groups were investigated and compared.

**RESULTS:**

The success rate of the placement in the downward group was significantly higher (95% vs. 75%, P< 0.001) and the average time for the successful placement was shortened (23 ± 5.91 min vs. 26 ± 5.49 min, P= 0.025) than that in the conventional group. No treatment-related adverse reactions occurred in either group, and there were also no significant differences in vital sign changes.

**CONCLUSIONS:**

The use of the downward tract adherence method in the manual bedside placement of postpyloric tubes for the intensive care patients at the bedside has a higher success rate, effectivity and safety.

## 1. Introduction

Critically ill patients admitted to the intensive care unit (ICU) are a high-risk group for malnutrition, with reported prevalence rates ranging from 38% to 78% [1]. Malnutrition is associated with muscle atrophy, prolonged ventilation time, prolonged ICU hospitalization, and increased risk of infection and death [2]. During the hospitalization of critical care patients in the intensive care unit (ICU), timely enteral nutrition support is key to improving patients’ prognosis. Enteral nutrition is superior to intravenous nutrition in terms of infection incidence, hospital stay, and medical expenses [3, 4, 5]. Due to the frequent occurrence of gastrointestinal dysfunction or failure in many critically ill patients, indwelling a gastrointestinal tube for feeding can easily lead to gastric retention, vomiting, and aspiration, and may even lead to aspiration pneumonia [6]. It is also recommended to start enteral nutrition as early as possible to protect the structure and secretion function of endothelial cells, and maintain immunity [7]. Intensive care patients who cannot tolerate gastrointestinal nutrition can receive effective enteral nutrition through nasogastric tubes for feeding [8, 9, 10, 11]. The methods for placing enteral nutrition tubes (EFTs) after the pylorus include endoscopy, fluorescence microscopy, ultrasound assistance, and electromagnetic guidance. However, EFT can also be placed blindly without auxiliary equipment. The success rate of fluorescence microscopy and endoscopy is relatively high [12]. However, they may not be suitable for patients with hemodynamic instability or severe respiratory failure, who cannot be transported outside the intensive care unit. Bedside blinding is usually used for critically ill patients because it is simple, minimally invasive, and cost-effective. However, placing the catheter in the correct position carries a significant risk of failure, which may lead to delayed enteral nutrition. Still, the complicated unassisted jejunostomy and low success rate of tube placement are often problems faced by healthcare workers in the process of implementation [13]. In recent years, the success rate of tube placement has been significantly improved and increased by using the double guide-wire placement method [14], the gas injection method [15], and the passive waiting method [16, 17]. There is relatively little research on the operational technical requirements during the surgical process [18]. In this study, the authors investigated the effectiveness of using downward tract adherence method when manually placing the posterior pyloric duct beside the bed in ICU patients. Our results showed that the use of downward tract adherence method for manually placing the posterior pyloric tube at the bedside for critically ill patients has a high success rate, effectiveness, and safety. To our knowledge, this is the first study that presented the advantage of downward tract adherence method for manually placing the posterior pyloric tube at the bedside for critically ill patients.

## 2. Methods

### 2.1 General information

The researchers performed a single-center prospective chart review of critically ill patients admitted to the ICU in a Provincial level three grade A hospital. All experimental protocols were approved by The Second Affiliated Hospital of Wenzhou Medical University (No. LCKY2020-370). Written informed consent forms were obtained from each patient or their next of kin. All methods were performed in accordance with the 1964 Declaration of Helsinki and later amendments.

**Figure 1.**
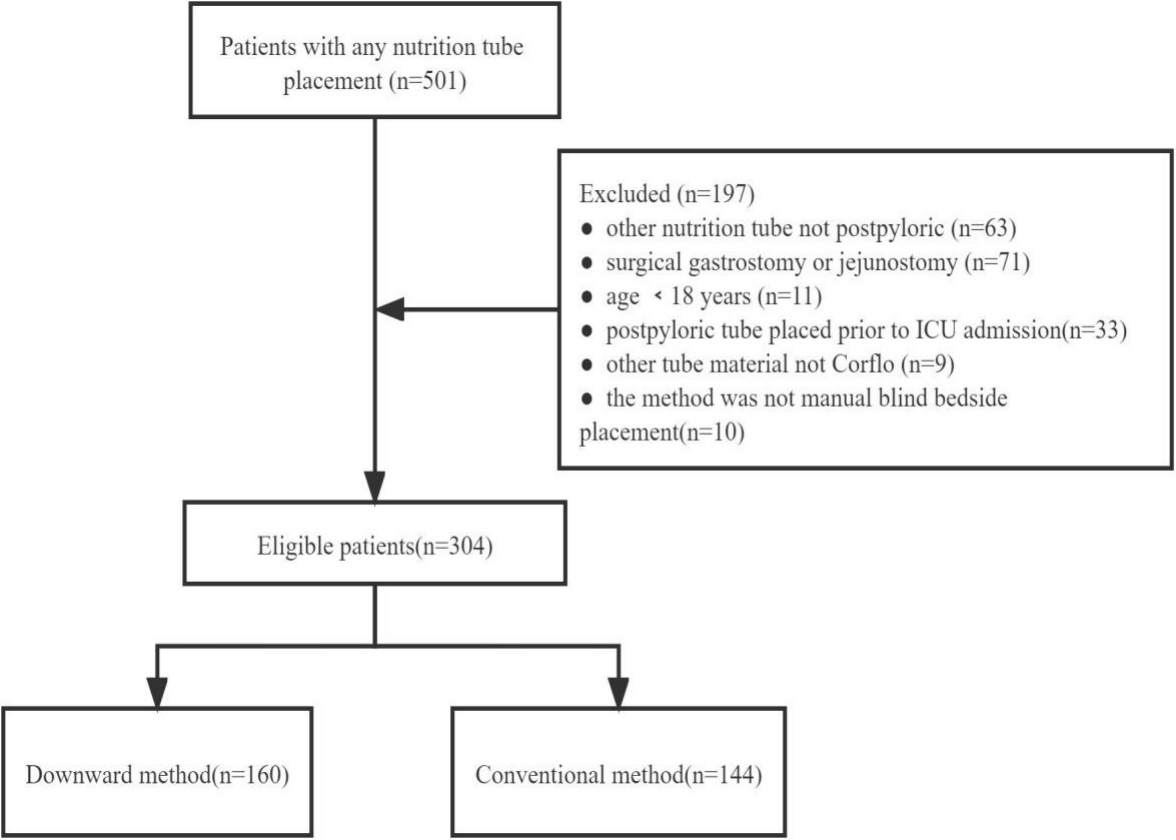
CONSORT flow diagram.

Inclusion criteria: 18 years of age or older, stay in the ICU more than 48 hours, first time receive the blind bedside postpyloric tube placement, the material is American Corflo “bullet” nasointestinal tube. Exclusion criteria: patients used other kinds of nasointestinal tubes; patients after gastrostomy or jejunostomy surgery; patients with postpyloric tube prior to ICU admission; younger than 18 years; not blind bedside placement; nutrition tube that in stomach. Three hundred and four consecutive patients were identified after screening the inclusion and exclusion criteria (Fig. 1) and were separated into two groups according the placement methods that were put into use. One group use the conventional method to perform the postpyloric tube and the other group applied the downward tract adherence method.

### 2.2 Materials and participants

#### 2.2.1 Materials

An American Corflo-10FR single-lumen gastrointestinal nutrition tube, a syringe (50 ml), a stethoscope, a treatment bowl (200 ml of water inside), a pair of sterile gloves, a strip of 3M adhesive tape and pH test paper, and so on were required to perform the experiments.

#### 2.2.2 Medical workers

There were five operators: one associate chief physician, one associate chief nurse practitioner, one attending physician, and two supervising nurse practitioners. All of them were members of the enteral nutrition sub-specialty team in our department and passed the systematic training on knowledge related to nasojejunal tube placement.

### 2.3 Tube placement method

The advantages, methods, time frame, and other precautions concerning enteral nutrition by nasal enteral tubes and the method of cooperation were explained to the awake patients before the tube placement. The patients’ stomachs were emptied at least 4 hours in advance. The lateral orifice of the nasoenteric tube was closed to ensure that the guide-wire connector remained firm during intubation. In the treatment bowl, 200 ml of saline was poured. The nasogastric tube was placed for immersion, and 50 ml of saline was injected into the lumen through a syringe; the outer end of the product and the inner lumen were treated with lubricant.

In the conventional group, the catheter was first placed into the stomach. The patients were seated in the right semi-sitting position, and the tube was placed into the stomach. The stomach of the patients was filled with 10 ml/kg of air, and the tube was delivered to the duodenum, with the operator maintaining a gentle, uninterrupted propulsive force. If the resistance is obvious, the catheter is relaxed so that it automatically retracts, and the catheter continues to be placed when it no longer retracts or when the resistance decreases. To initially confirm the position of the catheter, the catheter is placed on a scale of 110–120 cm. Then the guidewire is withdrawn, and the catheter is fixed.

**Figure 2.**
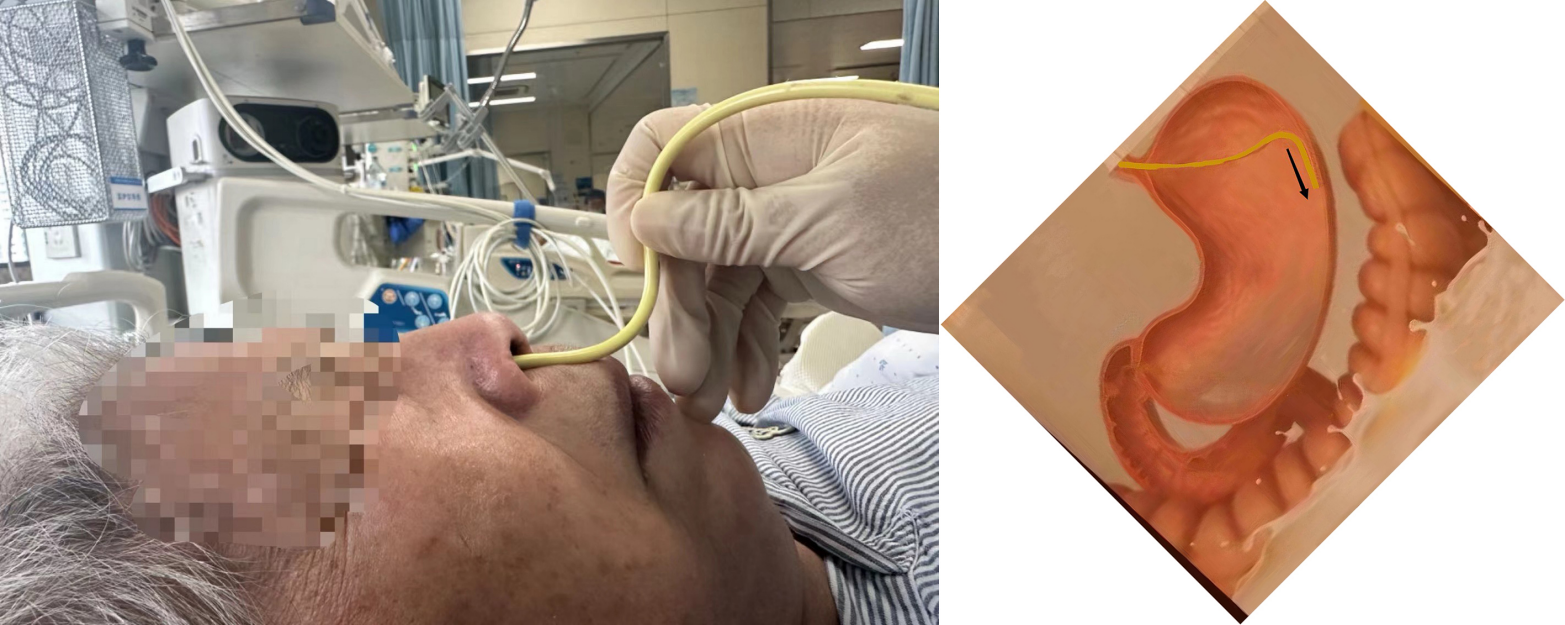
DONWARD tract adherence method.

In the downward group, a nasal tube was placed into the stomach of the patients. During the procedure, the patients were seated in the right semi-sitting position. The left hand of the operator held the end of the nasointestinal tube, while the right hand of the operator fetched the top of the nasointestinal tube like holding a pen and pressed the nasointestinal tube as far down as possible against the outer wall of the nasal cavity (Fig. 2). The tube was slowly advanced in accordance with the breathing rhythm of the patients, without actively inserting the tube downward. The operator took the inspiratory phase of the patients to insert the tube, 1–2 cm each time, and looked for the inhalation feeling so that the nasointestinal tube was inserted against the stomach wall. If resistance was felt at 75–90 cm of insertion, a syringe could be used to slowly inject gas while the nasogastric tube was being inserted. The position of the nasogastric tube was confirmed by checking the movement of the guidewire in the nutrient tube at 75, 80, 85, and 90 cm. During the placement of the nasogastric tube from the stomach to the posterior pylorus, the downward tract adherence method should be used for placement, the hand holding the tube should not be loosened, and the propulsion force should not be interrupted so that the displacement of the head end of the tube is avoided. If the tube passes smoothly through the pylorus, the guide wire will enter and exit the tube smoothly or with little resistance. When the guidewire cannot be inserted or has a high resistance after 45 cm, it indicates the probability of the nasogastric tube coiling in the stomach being high, so the guidewire should be inserted for adjustment while withdrawing the tube. After confirming that the nutrition tube is not coiled in the stomach, the nasogastric tube is slowly delivered to 110–120 cm in accordance with the respiratory rhythm of the patients, and the position of the catheter is confirmed. Then, the guidewire is withdrawn, and the catheter is fixed.

The following methods are used for preliminary confirmation of the tube location:

1. Auscultation method: Sequentially auscultate the sound of air over water in the left upper abdomen (stomach), right lower abdomen (pylorus, duodenum), and left lower abdomen (duodenum, jejunum) and determine the location of the strongest point.
2. Suction technique: Pump back the digestive fluid, observe its color properties and determine the pH value. Because the intestinal fluid contains bile, the digestive fluid is usually yellow and clarified, and the pH value should be greater than 6.
3. Vacuum pumping method: It includes pumping back after injecting about 20 mL of air or water through the tube. If more turbid gastric fluid or air is pumped out, it usually indicates that the tip of the catheter is in the stomach. If the pumping is negative pressure or a small amount of golden yellow small intestine fluid is seen, the tip of the catheter has passed through the pylorus.
4. Resetting of the guidewire: The withdrawn guidewire is repositioned, and when the tube is coiled or folded back in the stomach, the guidewire cannot be placed, or the guidewire has high resistance, the catheter needs to be re-withdrawn while the guidewire is placed for adjustment [19].

In both groups, the guidewire was withdrawn, and the catheter was fixed after confirmation by three or more methods, and 30 ml of warm boiled water was injected into the catheter as a rinse. A bedside abdominal X-ray was performed to confirm whether the catheter had passed through the pylorus. Patients were kept under cardiac monitoring during the entire placement process, and their vital signs were closely observed and recorded.

### 2.4 Observation index

1. Success criteria: an additional bedside examination of abdominal X-ray was conducted immediately after the operation was completed, and the X-ray film result was the gold standard. The head end of the catheter located in the duodenum or jejunum indicates successful placement; otherwise, it is regarded as a failure.
2. Calculation of placement time: The head end of the nasointestinal tube enters the nasal cavity as the starting time, and the ending time is the withdrawal of the guidewire.
3. Adverse reactions: These include placement-related adverse reactions and adverse drug reactions. The changes of vital signs are closely observed. If the heart rate, respiration, and mean arterial pressure decrease or increase by > 20% and SpO2 decreases by 5% during the placement, the placement is suspended or stopped.

### 2.5 Statistical analysis

Statistical analysis was performed using SPSS version 19.0 statistical software. Continuous variables were expressed as mean ± standard deviation, and a t-test was used for comparison between groups. Counting data were expressed as frequency and rate (N, %) and compared by the chi-square test. P< 0.05 difference was statistically significant.

## 3. Results

### 3.1 The success rate of intubation

Patients over 18 years old, who were admitted to the ICU from July 2020 to June 2022 and hospitalized in the ICU longer than 48 hours, who were first performed with postpyloric tube placement in the ICU. Under the reviewing of the technology database information, we identified the patients admitted to the ICU who were given the medical orders of nutrition tube placement. In the downward group, there were 96 males and 64 females who were admitted to ICU, the average age was 53.46 ± 3.46 years, and the primary diseases included 76 cases of severe craniocerebral injury, 12 cases of severe pancreatitis, 28 cases of spinal cord injury with paraplegia, 12 cases of chronic obstructive pulmonary disease (COPD), 4 cases of motor neuron disease, and 28 other cases. In the conventional group, there were 76 males and 68 females between July 2020 and 31th May 2022, the average age was 56.54 ± 6.54 years, and the primary diseases included heavy cranial injury in 68 cases, severe pancreatitis in 16 cases, spinal cord injury with paraplegia in 24 cases, COPD in 12 cases, and 24 other cases (Table 1). During the nasojejunal placement, the conventional group was placed by the traditional air injection method and the downward group was placed by the downward tract adherence tube enteral nutrition, and they signed the informed consent form. The differences in gender, age, admission diagnosis, and mechanical ventilation between the two groups were not statistically significant (all P> 0.05).

**Table 1:** Clinical and demographic data

Variables		Group		P value
		Experimental group (n= 160)	Control group (n= 144)	
Age, years	Male, n(%)	56.97 ± 6.98	56.70 ± 7.10	0.748
Gender		96 (60)	76 (52.8)	0.205
	Female, n(%)	64 (40)	68 (47.2)	
Primary diagnosis, n (%)				
Severe cranioc erebral injury		76 (47.5)	68 (47.2)	0.527
Severe pancreatitis		12 (7.5)	16 (11.1)	0.187
Spinal cord injury with paraplegia		28 (17.5)	24 (16.7)	0.482
COPD		12 (7.5)	12 (8.3)	0.476
Amyotrophic lateral sclerosis		4 (2.5)	0 (0)	0.075
Others		28 (17.5)	24 (16.7)	0.485

Data are presented as n (%) or median with interquartile range.

In the downward group, the one-time placement success rate in 160 patients was 92.50% (148/160), and the placement success rate was achieved during the second attempt in four patients, with an overall success rate of 95.00% (152/160). In 4 cases, the catheter was still coiled or folded in the stomach after secondary placement, so it was placed under endoscopic guidance. The remaining 4 cases were severe traumatic brain injury with gastric paresis, and were automatically discharged during secondary placement, but the catheter could not be placed. In the conventional group, the total success rate of tube placement was 75.00% (108/144) among 144 patients, with 64.00% (92/144) for one-time tube placement and 16 cases for secondary tube placement. While comparing the success rate of cannulation in the two groups, the difference was found to be statistically significant (P< 0.05, Table 2).

**Table 2:** Comparison of the success rate of tube placement in the experimental and control groups

Group	Cases (N)	Success rate of tube placement (N, %)
Experimental group	160	152 (95.00%)
Control group	144	108 (75.00%)
χ⁢2	4.613	
P	0.032	

### 3.2 Mean time for placement

The time required for placement were recorded and compared. The average placement time for all patients is 24 ± 5.35 minutes. In the descending group, the average time spent on the descending tract adhesion method was 23 ± 5.91 minutes, while in the traditional group, the average time spent on the traditional method placement was 26 ± 5.49 minutes. The difference between the two groups was statistically significant (P< 0.05, Table 3).

**Table 3:** Comparison of the mean time taken for successful tube placement in the experimental and control groups

Group	Cases (N)	Placement time(min)
Experimental group	160	23 ± 5.91
Control group	144	26 ± 5.49
T	2.285	
P	0.025	

### 3.3 Adverse reactions

No drug-related adverse reactions were observed in all patients. Each patient undergoes cardiac monitoring and monitors and records changes in vital signs. Some patients experience an increase in heart rate during intubation, but not exceeding 30%, and it automatically subsides at the end of intubation. The blood pressure, heart rate, blood oxygen saturation, and respiratory rate of other patients did not show significant changes. The patient did not experience any complications such as suffocation, bleeding, allergies, or emphysema during or after the surgery.

## 4. Discussion and conclusion

Nutrition to patients in the ICU can be given by parenteral nutrition or enteral nutrition. In cases where enteral nutrition is available, transjejunal feeding is the preferred route for intensive care patients [20, 21]. In recent years, manual bedside placement of postpyloric tube has gradually become an important method for implementing postpyloric feeding in intensive care patients. To our best knowledge, different methods of nasointestinal tube placement have their own advantages, but each has unavoidable drawbacks. Most studies in the past have involved nasointestinal tube placement assisted by gastroscopy/X-ray [22, 23], which often requires appointment scheduling and staffing and involves the transfer of the patient to a specific site, which carries a risk of transport. The entire tube placement requires multi-department collaboration and is costly for the patient. There are also more hospitals in China that use the fulcrum spiral nasointestinal tube injection method [24, 25] or ultrasound-guided method [26, 27], which can be operated at the bedside, but the method is more complicated and the success rate is not high. This prospective, single-center study revealed that the bedside blind technique of downward tract intubation method for post-pyloric placement of nutrition tubes was effective along with time saving in critically ill patients. The overall success rate of post-pyloric placement was 95%, which was an encouraging result compared with the conventional method. In addition, the average consumed time of placement was less than the conventional method, which allows the implementer has more available chances of intubation to meet the guideline recommended golden window (48 h) for early EN [28, 29, 30].

According to Bing et al. [31], the active placement method of spiral nasointestinal tubes is relatively difficult and has a low success rate, and the success rate of spiral tube placement in patients with gastrodynamic dysfunction is only 57%. Major hospitals in China have introduced Corflo gastrointestinal nutrition tubes in the clinic since 2009 [32, 33]. The safety of the clinical application of Corflo gastrointestinal nutrition tubes has been proved in several downward studies at home and abroad, and the success rate of its placement is about 74%–94% [34], as reported in the existing literature, but the technique used for placement is less involved. In the present study, we have described the clinical experience of manual beside blind placement of postpyloric in the ICU patients. The new method indicates a stable and safe situation of patients, vital signs before and after intubation can be well controlled without serious adverse effects between the two groups. The risk of accidental entry into the trachea during tube placement can be detected and dealt with promptly by clinical observation (e.g., the catheter can be removed and reset if the patient coughs). The risk of inadvertent aspiration can be better avoided by good testing and positioning and confirming the position of the catheter before feeding. The possibility of tube blockage during feeding can also be reduced through close clinical observation, and standardized nursing procedures can be introduced to minimize complications. The overall expected medical risks are relatively small, and the overall safety is high. However, tube blockage may still occur in the feeding process, which requires strict clinical observation and standardized nursing to reduce complications.

In this study, the findings demonstrate that there is a substantial endorsing for downward tract adherence method among critical ill patients in ICU. It was observed that the conventional placement method was obstructed by the catheter being placed too quickly and the cephalic end being embedded in the gastric wall. The cephalic end of the catheter tends to deflect when it encounters resistance to retraction or relaxation, and the catheter is coiled in the stomach, making it difficult to pass through the pylorus leading to placement failure [35, 36]. The downward tract adherence method is less likely to detach from the stomach wall, and the tip of the tube can turn on its own when it meets resistance, improving the success rate of tube placement to a greater extent. This prospective study shows that the downward tract adherence method is a more effective and safer technique for manual bedside placement of postpyloric tubes in intensive care patients, which is worth being promoted in clinical practice.

### 4.1 Limitations

There are several limitations in this study and the promotion of generalizability could be limited. As the operation was only processed by the senior intensivists who were trained for a short period of time, the bias may exist while the procedure was performed by the junior intensivists. All physicians participated the study performed the procedure following the same guideline after completing the same training program. Therefore, we did not consider operator was a variable in this study. However, in different clinical institutes where different guideline was employed to instruct the physicians to perform the procedure, different results might be obtained. In order to improve the current situation, our team has conducted a series of studies on the optimal placement of ICU staff. As a newly introduced technology, it needs more time and experience to become a routine ICU training program for intensive care physicians in Chinese Mainland [30]. In addition, in current prospective studies, more than half of patients were diagnosed with traumatic brain injury, which may be a potentially relevant fact affecting the results, although both groups of patients did not show statistical significance in terms of gender, age, admission diagnosis, and baseline mechanical ventilation. In addition, this prospective study did not analyze the effectiveness and economic benefits of the prognosis. Last but not least, all patients used the postpyloric tube called American Corflo “bullet” nasointestinal tube. The result may be different when other kinds of tubes are used.

### 4.2 Recommendations

Further studies are needed for a comprehensive evaluation to promote the application of downward tract adherence method in bedside intubation technology. Second, larger-scale, multi-center studies among different patients assessing the clinical utility of downward method as well as analyzing financial costs are needed. Third, more studies focus on developing a training course to improve the overall success rate of downward tract adherence method can also be significant for clinically medical workers in future.

## Ethical approval

All experimental protocols were approved by The Second Affiliated Hospital of Wenzhou Medical University (number: LCKY2020-370; date: 13 November 2020).

## Informed consent

Written informed consent was obtained from all participants after clarification of the study objectives and activities.

## Funding

None to report.

## Author contributions

Conceptualization: Weiwei Cai; Project administration: Zhebing Lin; Data curation: Zhisu Li; Software: Zanhua Zhang, Fang He, Zhebing Lin, Zhisu Li, Fei Xiang, and Weiwei Cai; Writing-original draft: Zanhua Zhang; Writing-review & editing: Fang He and Fei Xiang. All authors have read the manuscript and gave their final approval of the version to be published.

## Availability of data and materials

The datasets used and analysed during the current study are available from the corresponding author on reasonable request.
